# Single versus Double Plate Fixation in Condylar Neck Fractures: Clinical Results and Biomechanics Simulation

**DOI:** 10.3390/bioengineering11070704

**Published:** 2024-07-11

**Authors:** Chien-Chung Chen, Ting-Han Chiu, Cheng-Yu Yan, Ya-Pei Hou, Ting-Sheng Lin

**Affiliations:** 1 Department of Plastic and Reconstructive Surgery, E-Da Hospital, Kaohsiung City 82445, Taiwan; chenmed.tw@gmail.com (C.-C.C.); jasonchiu95@gmail.com (T.-H.C.); 2 College of Medicine, I-Shou University, Kaohsiung City 82445, Taiwan; 3 Department of Biomedical Engineering, I-Shou University, Kaohsiung City 82445, Taiwan; betty032533@gmail.com; 4 Biotechnology and Biochemical Engineering Center, I-Shou University, Kaohsiung City 82445, Taiwan; yapei0110@isu.edu.tw

**Keywords:** condyle fracture, mandible, fixation, mini-plates, finite element analysis

## Abstract

The open reduction of mandibular condyle neck fractures is difficult due to the limited surgical field and complex facial nerve structures. The most effective fixation method for narrow fractured segments is debated as standard double four-hole plate fixation is often not feasible. This research compared bone stability and force resistance between single-long-plate and double-short-plate fixations using clinical outcomes, a Sawbones mandible model, and finite element analysis. In patients with condyle neck fractures, nine were fixed with single-long-plate and twelve with double-short-plate fixations, with no significant differences in malocclusion and facial palsy rates. In compression tests with a Sawbones model, displacements in the posterior part were similar in both fixation groups. In contrast, the anterior part had significantly higher displacements in the single-long-plate group. Finite element analysis showed higher displacements in both anterior and posterior parts in the single-plate group compared to the double-short-plate group. Maximum stresses were at the second screw hole in single-long-plate fixation and the turning point of the upper plate at the condyle neck in double-short-plate fixation. Double-short-plate fixations demonstrated better stability and force resistance than single-long-plate fixations.

## 1. Introduction

Managing the condylar fractures of the mandible remains a tremendous technical challenge, even for experienced surgeons. Its complex anatomy and biophysical characteristics continue to perplex surgeons and prevent them from obtaining the best treatment approach, resulting in endless controversies between open reduction and closed treatment [1,2]. Over the past few decades, emerging evidence in the literature has supported the superiority in recovery time and outcomes with open reductions for severely displaced condylar mandibular fractures [3,4,5]. However, surgical technique, neurological damage, and the various inconsistent classification systems adopted in current practice have caused severe confusion in defining the specific fracture sites and patterns to provide a reliable management approach.

On the other hand, the plating and fixation options for individual fracture sites pose significant challenges. Two-plate fixation has been established in experimental and clinical studies [6,7,8] and is generally accepted for fixing sub-condylar mandibular fractures. However, for the narrowest portion of the condylar “neck” in mandibular fractures, two-plate fixation is often technically infeasible given the limited surgical field and bone width for manipulation. Because of the limited options for fixation suggested by mandibular condylar fractures and the lack of clinical consensus, we chose to examine whether the application of either a single long plate or two short plates for fixation would produce superior bone stability. 

Finite element analysis (FEA) is a crucial numerical method utilized in biomechanical research, specifically in orthopedics and plastic surgery, to predict and optimize the mechanical behavior of biological tissues, implants, and surgical procedures [9,10,11]. This method commonly aids in understanding the performance of orthopedic devices and optimizing implant design, yet it also helps in analyzing soft tissue mechanics and assessing the effects of surgical interventions in plastic surgery. FEA enables the simulation of surgical procedures and reduces the need for expensive and time-consuming experiments to eventually develop safer and more effective treatments in these medical fields. Therefore, in this study, we used a conventional compression test using a Sawbones model and FEA simulation were conducted to analyze the proposed plating pattern simulations and resulting stability. This study aimed to determine the appropriate fixation for condylar neck fractures with clinical outcomes and simulations of different plating methods on a Sawbones model and finite element analysis to compare its stability and relative bone displacement under different forces.

## 2. Materials and Methods

**Figure 1:**
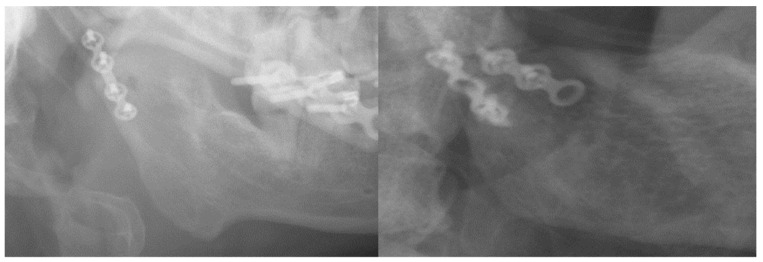
Demonstration of single-long-plate and double-short-plate fixations on a panoramic X-ray.

A retrospective review of all mandibular neck fractures from 2013 to 2022 who visited a single surgeon’s clinic at E-Da Hospital for evaluation and intervention was conducted. The mandibular fracture sites, the conservative or surgical treatment adopted, the surgical approach, and the fixation methods of either single long plate or double short plates (as demonstrated in Figure 1) were documented. The surgical outcomes of occlusion and facial palsy were analyzed.

**Figure 2:**
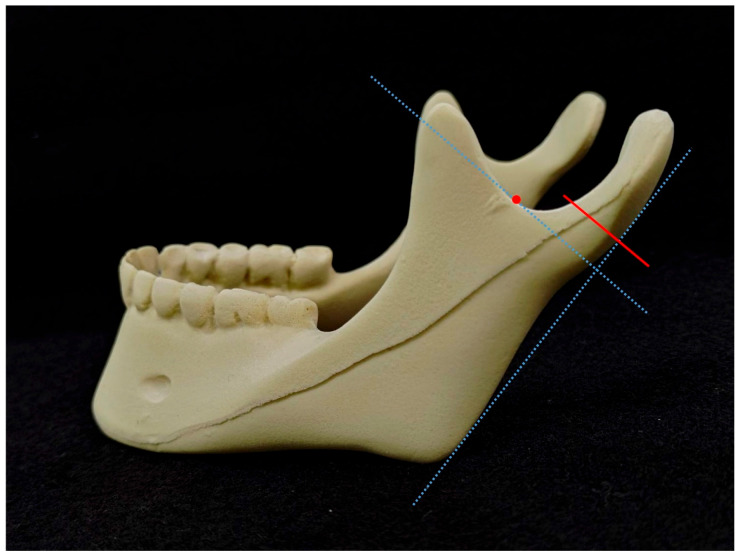
Sawbones model and design.

**Figure 3:**
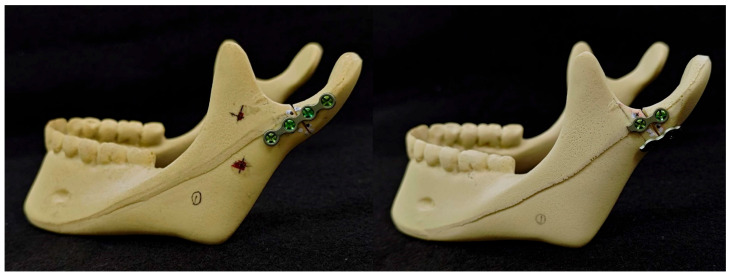
Demonstration of single-long-plate and double-short-plate fixation on Sawbones models.

**Figure 4:**
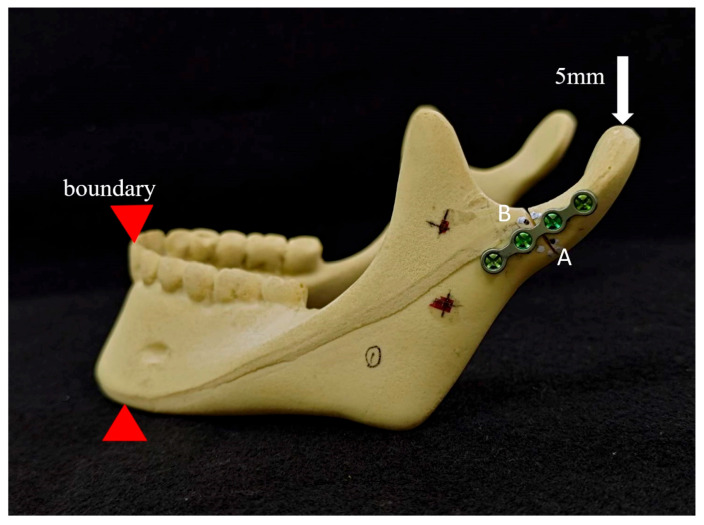
Demonstration of compression test of Sawbones model.

In this study, an in vitro mandible model (#1337-3, Mandible with Teeth, Foam Cortical Shell and Cancellous Inner Material, Sawbones, Pacific Research Laboratories Inc., Washington, DC, USA) was utilized in the biomechanical experiments. This synthetic bone model has been previously documented in the literature as a viable substitute for bones in orthopedic medical devices and biomechanical experiments [12,13]. The condylar neck fracture was defined as described in the AO-CMF classification [14]: an imaginary line of the lateral mandibular border was drawn, and a perpendicular line crossing the lowest point of the mandibular sigmoid notch was thus designed; the latter line was then translated upwards 2 mm to form the cutline (marked in red) on the model for fracture simulation (Figure 2). Two groups of different fixation methods were demonstrated in Figure 3 as follows: single-long-plate groups (13 samples) were fixed with a four-hole mini-plate and four screws (Leibinger 2.0 mm miniplate system), while the double-short-plate groups (13 samples) were fixed with two parallel two-hole mini-plates and four screws. The plates were placed perpendicular to the fracture line created. We then performed the compression biomechanical experiment by utilizing the universal testing machine of MTS Q-test/10 (MTS Systems Co., Evanston, IL, USA) to evaluate the resistance of the fixed fracture models. Two reference points were defined and marked on the Sawbones model, with point A on the fracture line’s posterior side (condyle part) and point B on the anterior side (jaw part). The model was held and fixed at the incisors, and then the jig was applied to the mandibular condyle to form a consistent downward displacement of 5 mm (Figure 4). We then estimated the displacements of points A and B using the three-dimensional digital image correlation [15] method to compare the coordinates before and after the displacement-controlled compression test. 

The same Sawbones model was adopted as the reference for the finite element analysis. We first performed a computed tomography scan for the Sawbones model, and the images were integrated into the medical image processing system for three-dimensional reconstructions. During the process, the coordinates of each section were acquired and then processed with computer-aided design software (Creo Parametric 9.0, Parametric Technology Co., Boston, MA, USA) to form the complete three-dimensional model. The simulation of plates and screws was also identical, as described in our previous models. We then set the parameters of the boundaries and the applied forces as the exact numbers as in the mechanical experiments. The Young’s modulus of the Sawbones model was 13,700 MPa, and the screws and plates were 110,000 MPa, while the Poisson’s ratio was 0.3 and 0.34, respectively. We then analyzed the displacement around the surface of the fracture line and compared the results with the virtual models.

All statistical analyses were performed with IBM SPSS Statistics 25 for Mac (Armonk, New York, NY, USA). The associations of the clinical outcomes between the two different plating pattern groups were performed with the Fisher’s exact test for categorical variables and of the displacement in the biomechanical simulation with the Mann–Whitney U-test for continuous variables. The statistically significance level of this study were set to *p* = 0.05.

## 3. Results

In the condyle neck fractures we included, nine were fixed with single-long-plate (four-hole-four-screws) and twelve with double-short-plate (three-hole-two-screws) fixations. Eighteen out of all twenty-one patients (85.7%) were treated using a pre-auricular approach. No difference was found in malocclusion rates (*p* = 1.00) and facial palsy rates (*p* = 1.00), as shown in Table 1.

**Figure 5:**
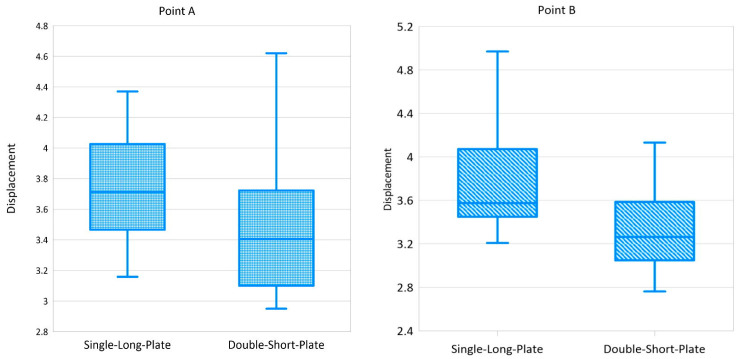
Interquartile range results of displacement of fracture site (**left**: point A; **right**: point B).

In the compression test of the Sawbones model, the displacements of the posterior point A were comparable in both fixation groups (*p* = 0.112), though slightly more in the single-long-plate group. In contrast, the displacements of the anterior point B were significantly greater in the single-long-plate group (*p* = 0.043) (Table 2). Interquartile range results are also shown in Figure 5.

**Figure 6:**
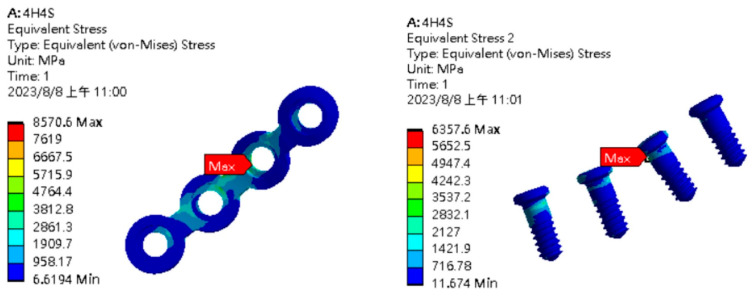
Results of finite element analysis for single-long-plate fixation (**left**: plate; **right**: screw).

**Figure 7:**
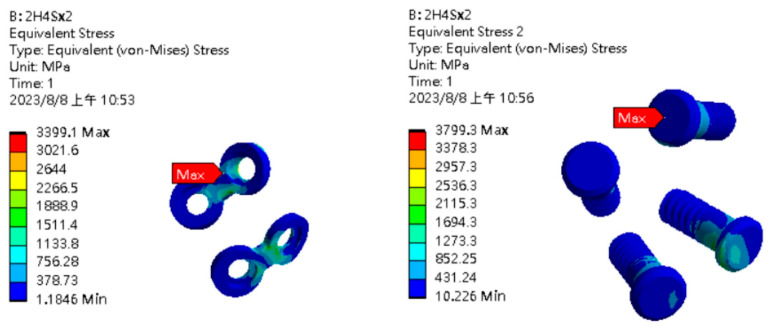
Results of finite element analysis for double-short-plate fixation (**left**: plate; **right**: screw).

In the finite element analysis, the displacements of points A and B were 5.37 mm and 5.35 mm in the single-long-plate group but were 4.35 mm and 4.17 mm in the double-short-plate group. The maximum von Mises stress in the single-long-plate fixation was found at the dorsal side of the second screw hole, with the stress force of 8570.6 MPa and 6357.6 MPa at the plate and screw, respectively (Figure 6). However, the maximum von Mises stress in the double-short-plate fixation was found at the turning point of the upper plate at the condyle neck at 3399.1 MPa for the plate and 3799.3 MPa for the screw (Figure 7).

## 4. Discussion

Condylar process fractures consisted of a considerable 17.5 to 50% of all mandibular fractures [16], and the severe functional consequences and difficulty in surgical reductions deserve greater attention in current craniofacial reconstructions. Given the frequently encountered technical difficulty in managing such fracture type, the authors came up with an innovative approach to replace the standard long-plate fixation with a double shorter-plate fixation method in order to overcome this surgical challenge. To our up-to-date knowledge, previous research involving similar methods has yet to be reported. The authors were able to conclude that patients fixed with a single long plate and with double short plates resulted in comparable clinical outcomes, as well as achieving better bone stability in both biomechanical compression tests and finite element analysis. 

Although numerous studies regarding the treatment outcomes of condylar fractures have been conducted over the years, controversy remains as those results were not directly comparable owing to the adoption of a disparate array of classification systems [17]. Some of the most frequently adopted classifications in clinical practice are based on different perspectives, ranging from pure anatomically based systems [18,19] to fracture characteristics-based ones [20] to a combination of both [14,21,22]. Experts have now advocated the ubiquitous use of the AO-CMF system to solve disputes and provide specific guides to each subtype of mandibular fracture. 

According to the AO-CMF classification, the “neck” is the narrowest portion of the condyle. Its complex anatomy with adjacent facial nerve branches and the parotid gland has also made surgical reduction extremely challenging to identify critical structures directly and to apply proper fixation within such limited working space. Various surgical approaches have been proposed to facilitate the open reduction of the mandibular condyle, including pre-auricular, retro-auricular, and retro-mandibular (retro-glandular, ante-glandular, or trans-parotid gland) approaches. Theoretically, the trans-parotid gland approach reaches directly to the fracture site, providing a better surgical field and less nerve traction than other methods. However, delicate nerve dissection techniques must be adopted to avoid injuring the nearby facial nerve trunk [23]. So far, no substantial evidence has been found in the literature suggesting the best approach for condylar neck fractures. 

Regardless of the surgical approach, a limited surgical field to operate over a rather complicated anatomical structure is inevitable during condylar neck reductions. Therefore, choosing the appropriate implant hardware and fixation method becomes critical to achieving proper reduction outcomes for such fractures. The two-plate fixation is generally accepted as the adequate rigid fixation method for mandibular fractures over the sub-condylar area. This method has been demonstrated through in vitro fine elemental simulations^4^ and biomechanical models [7] as well as in an in vivo clinical study with 831 patients on the complications of hardware failure, screw loosening, and malocclusion [8]. In theory, multiple plate fixation provides better strength and stability as the weight bearing is distributed along different axials. A recent study has shown that trapezoidal plates induce minimal strain on the cortical bone and best resist displacement [24]. However, applying two plates, even in a parallel form or Y-shape, is sometimes unfeasible at the narrowest region of the condylar neck. In such cases, applying two plates with merely two holes (simulating the form of a delta plate) crossing the fracture site may be a potential solution to resist the torsional forces better and retain adequate rigidity without requiring extensive dissection. Previous studies have also shown superior fixation rigidity with multiple shorter plates to fewer longer plates [25]. 

This study demonstrated that the proposed two-plate-two-hole technique improved strength to withstand compression forces compared to a single-plate-four-hole fixation. Notably, significantly less displacement was found at the anterior border of the condylar neck fracture in the compression test, indicating that the condyle was less likely to suffer from recurrent condylar collapse after such double-plate fixations. Our results suggested that shorter plates did not compromise the rigidity as long as the shearing forces were counteracted well enough. Based on the finite element analysis results, we observed that greater stresses were predominantly concentrated on the bone screws and bone plate near the fracture location. From this observation, we concluded that the forces were primarily distributed among the bone screws in proximity to the fracture site. As a result, stresses on the bone screws and plates in the double-two-hole configuration were reduced compared to the conventional four-hole plate fixation method. This phenomenon might be attributed to the fact that the conventional four-hole plate has less force distribution capability among the screws at the ends of the plate, leading to greater stress concentrations on the middle two screws [10]. The compression force generated in our simulation reached up to 100 N, exceeding 60% of the maximum natural mastication forces required for chewing during the healing process of mandibular fractures [26,27], indicating adequate resistance under such plate fixation patterns. Under this hypothesis, we could avoid extensive dissection and tissue destruction only to reach longer longitudinal space for a four-hole plate fixation. If further combined with a trans-parotid approach, the disturbance to the surrounding tissues could be minimized to reduce the chance of injuring facial nerve branches during traction for optimizing the surgical field.

This study is the first in vitro model analyzing the designed plating pattern corresponding to the dilemma frequently encountered in actual surgical practice. Nowadays, minimally invasive approaches to reduce possible damage to the tissues and adjacent facial nerves have gained popularity in managing sub-condylar neck fractures of the mandible. The authors were thus encouraged to find a simple yet effective plating method combining the emerging trans-parotid approach and minimal dissections to incorporate into our surgical protocol. However, it is essential to note that mandible stability and forces to withstand during the healing process are not solely related to bony structures alone but are considerably associated with the masticatory muscles and surrounding soft tissues, which could potentially alter the physical environment simulated by our Sawbones models. Also, the mechanical characteristics of the Sawbones models were not identical to human bones. However, the mastication force during the healing process of mandibular fractures was reduced to about 60 percent of the normal functioning forces [27]. Despite the limitations of the model used in this study, our results were still valuable and may provide helpful insight into the medical practice of managing mandibular condyle fractures. 

Additionally, due to the limitation of the mechanical testing system we utilized in the force resistance experiments, the force generated could only be calculated through the designated displacement after manipulation, which could result in less precision in the mechanical characteristics and behavior of the actual model. Furthermore, within the finite element analysis model employed in this study, the mechanical properties of the mandibular bone are exclusively defined as those of the cortical bone. Consequently, there excessively high stress and displacement values may occur. Despite this limitation, such numerical results from the stress analysis can still be used as a specific qualitative reference [9]. Nevertheless, its actual application in patients with mandibular fractures will require further study. The authors intend to design a prospective control case series for managing condyle neck fractures with an inclusion of a larger patient sample size. Moreover, more objective examinations of postoperative temporomandibular joint functional tests and oral function exams should be adopted into our standard follow-up practice [28,29,30], as well as a more detailed information about postoperative pain score, recovery time to normal oral diet, and subjective appearance satisfaction. We will aim to refer to previous research to improve our investigation of the fracture fixation methods discussed in this study. In future studies, we will also attempt additional simulations of various double plates or other plating patterns on this Sawbones model to directly compare those proposed fixation methods mentioned in the current literature.

## 5. Conclusions

It is important to clarify the proper fixation method for specific mandibular condylar fractures to reduce confusion in future studies and clinical practice. Our experimental and clinical study has shown that the two-plate-two-hole fixation method provides comparable outcomes with smaller surgical field dissections. We believe that this method could reasonably facilitate and encourage surgeons to perform proper reduction and fixation in mandibular condylar neck fractures.

## Figures and Tables

**Table 1:** Clinical outcomes between condyle neck fractures fixed with a single long plate and double short plates.

	Single-Long-Plate	Double-Short-Plate	*p* Value
Malocclusion	11.1% (1/9)	8.3% (1/12)	*p* = 1.00
Transient facial palsy	33.3% (3/9)	33.3% (4/12)	*p* = 1.00

**Table 2:** Displacement results of compression test using Sawbones model (unit: mm).

	Point A		Point B	
Group	Single-Long-Plate	Double-Short-Plate	Single-Long-Plate	Double-Short-Plate
Mean	3.838	3.314	3.867	3.157
S.D.	0.603	0.761	0.678	0.754
*p* Value	*p* = 0.112		*p* = 0.043	

S.D.: standard deviation.

## Data Availability

The original contributions presented in the study are included in the article; further inquiries can be directed to the corresponding author/s.
